# Supplementary material for: iDiabetes platform—enhanced phenotyping of patients with diabetes for precision diagnosis, prognosis and treatment: study protocol for a cluster-randomised controlled study in Tayside, Scotland
Source: BMJ Open. 2024 Nov 28;14(11):e086594. doi: 10.1136/bmjopen-2024-086594 (PMC11605812; doi:10.1136/bmjopen-2024-086594)
Supplement: online supplemental file 2 [file bmjopen-14-11-s002.pdf]

What happens to my health data?

In this research study we will use information from your medical records. We will only use information that we need for the research study. We will let very few people know your name or contact details, and only if they really need it for this study.

Everyone involved in this study will keep your data safe and secure. We will also follow all privacy rules. At the end of the study we will save some of the data in case we need to check it and for future research. We will make sure no-one can work out who you are from the reports we write. The information on the website tells you more about this.

What happens to my blood samples?

Your blood samples will be processed as usual within the NHS labs and the results used to inform your clinical care and make *i*Diabetes recommendations.

NHS blood samples are usually destroyed after seven days. If you are happy for us to store your left-over anonymised samples and health data for research to improve diabetes care, then we would encourage you to join the SHARE register (registerforshare.org).

If you do not wish to be involved

You do not have to take part and the standard of care you receive will not be affected. You can opt out of *i*Diabetes at any time without giving a reason. You can do this at your diabetes review by speaking to your doctor or nurse. You can also contact the *i*Diabetes team via the website or by phone.

If you'd like more information

You can find out more about *i*Diabetes, the blood tests and how recommendations are made on our website.

Research team contacts

w w w.iDiabetes.org.uk

iDiabetes@dundee.ac.uk

01382 388 008

This research is funded by the Chief Scientist Office, Scottish Government and Sponsored by the University of Dundee. It is being run by researchers from the Universities of Dundee and Aberdeen and NHS Tayside. It has been reviewed by an NHS Research Ethics Committee and has raised no objections from the point of view of medical ethics.

iDiabetes Plus, Patient Flyer, V3, 28th June 2023 (adapted version - images of non-patient individuals in the original version removed)

23672

*i*Diabetes

Precision diabetes care

*i*Diabetes

Precision diabetes care

NHS Tayside is testing an enhanced care approach for people with diabetes. *i*Diabetes, which stands for intelligent diabetes, uses your medical records with blood tests and computer algorithms to help your diabetes care team offer you more precise treatment. Any recommendations made will be specific to you, based on your individual risks and likelihood of benefit, rather than the current one size fits all approach.

We are very excited about this new approach but to show that it is better than the current care you receive, and is good value for money, we need to introduce it as part of a research study. Across Tayside, patients will receive ongoing standard diabetes care or an *i*Diabetes care approach.

How does *i*Diabetes work?

You attend for your diabetes review.

Additional blood tests are requested to check for markers and gene variations.

Blood samples are tested as usual by NHS Laboratories.

Results are analysed using information about you such as your age and type of diabetes.

***i*Diabetes asks:**

- Do you have an increased risk of heart or liver disease?
- Is your diabetes control sub-optimal?
- Should you be on additional treatment?

Results are sent to your health care team within four weeks.

***i*Diabetes may recommend:**

- A change to a new medication
- Lifestyle changes
- Further tests, such as a heart or liver scan
- No changes

*i*Diabetes only makes recommendations. You and your doctor or nurse can agree on what is the right treatment for you.

We will follow how you get on for up to 15 years through your health records.

What are gene variations and why do we want to test for them?

Genes differ between people and these variations explain in part how we differ from each other. We will measure your gene variations to help improve how we treat your diabetes and how we estimate your diabetes related longer term risks. Specifically, for this study we will use these variations to generate two risk scores. The first one is for your risk of type 1 diabetes, which we'll use to help confirm your type of diabetes. The second one is for heart disease risk, which we will combine with other risk factors to help decide if you need treatment that reduces the risk of heart disease. As we are not undertaking tests for individual conditions there are no implications for health or life insurance.

You will be asked at your appointment to confirm you agree to these genetic tests. You can still be included in the study if you do not agree to the genetic tests.

What does this mean for me?

Your GP practice is taking part and has been selected at random (like tossing a coin) to implement *i*Diabetes Plus care. This will start when you attend your next diabetes review.

Can I see my results and treatment recommendations?

You will be able to see your results and tailored information in My Diabetes My Way. This 'patient portal' lets you find out all about your diabetes, and diabetes in general, and we encourage you to sign up to this. (mydiabetesmyway.scot.nhs.uk)

Follow up recommendations will occur when there is a change in your risk. For example, if you are treated with insulin and your risk of low blood sugar increases, *i*Diabetes will flag this risk to your healthcare team.

University of Dundee
UNIVERSITY OF ABERDEEN
NHS Education for Scotland
NHS Tayside

The iDiabetes Platform: the views and experiences of health professionals and patients

Consent form: Patient interviews Participant ID_________

Chief Investigator: Prof Ewan Pearson, Professor of Diabetic Medicine

Sponsor: University of Dundee

Please initial box for each statement below:

Statement	Initials
1. I have read and understood the information sheet [version__ /date__] for the research project entitled "The iDiabetes Platform: the views and experiences of health professionals and patients".	
2. I have had enough time to consider the information and to ask questions, and I am happy with any answers I have been given.	
3. I understand that my participation in the interview(s) is voluntary and that I am free to withdraw my participation at any time, without giving any reasons. I know this will not affect my care or legal rights.	
4. I agree to the researcher audio-recording the session.	
5. I understand that data collected during the study will be looked at by the research team, and that all information will remain anonymous and confidential, and that no personal information will be used which may identify me or any other people in the final report or scientific publications.	
6. I understand that all data collected (e.g. audio-recordings) will be stored in a secure University server and line with current Data Protection regulations.	
7. I understand that data collected during the study may be looked at by individuals from regulatory authorities who have a duty to monitor the quality of the research, and I give permission for this.	
8. I understand that if any disclosures are made during the sessions that suggest malpractice, misconduct, or that someone is in danger of harm, this information will be shared with the appropriate personnel.	
9. I agree to take part in this research study.	

Name of participant (capitals) Date Signature

_______________________ __________ _______________

Name of person taking consent (capitals) Date Signature

_______________________ __________ _______________

The iDiabetes Platform: the views and experiences of health professionals and patients

Consent form: Staff interviews Participant ID__________

Chief Investigator: Prof Ewan Pearson, Professor of Diabetic Medicine

Sponsor: University of Dundee

Please initial box for each statement below:

Statement	Initials
1. I have read and understood the information sheet [version/date] for the research project entitled "The iDiabetes Platform: the views and experiences of health professionals and patients".	
2. I have had enough time to consider the information and to ask questions, and I am happy with any answers I have been given.	
3. I understand that my participation in the interview(s) is voluntary and that I am free to withdraw my participation at any time, without giving any reasons. I know this will not affect my care or legal rights.	
4. I agree to the researcher audio-recording the session.	
5. I understand that data collected during the study will be looked at by the research team, and that all information will remain anonymous and confidential, and that no personal information will be used which may identify me or any other people in the final report or scientific publications.	
6. I understand that all data collected (e.g. audio-recordings) will be stored in a secure University server and line with current Data Protection regulations.	
7. I understand that data collected during the study may be looked at by individuals from regulatory authorities who have a duty to monitor the quality of the research, and I give permission for this.	
8. I understand that if any disclosures are made during the sessions that suggest malpractice, misconduct, or that someone is in danger of harm, this information will be shared with the appropriate personnel.	
9. I agree to take part in this research study.	

Name of participant (capitals) Date Signature

_______________________ ________ ______________

Name of person taking consent (capitals) Date Signature

_______________________ ________ ______________

iDiabetes: Echo scan

Consent form: iDiabetes: Echo Scan   Participant ID_________

Chief Investigator: Prof Ewan Pearson, Professor of Diabetic Medicine

Sponsor: University of Dundee

Please initial box for each statement below:

Statement	Initials
1. I have read and understood the participant information sheet [version__ /date__] for the research project entitled "iDiabetes Study: Echo Scan".	
2. I have had enough time to consider the information and to ask questions, and I am happy with any answers I have been given.	
3. I understand that my participation in the study is voluntary and that I am free to withdraw my participation at any time, without giving any reasons. I know this will not affect my care or legal rights.	
4. I agree to have an echo scan using US2Ai software.	
5. I understand that data collected during the study will be looked at by the research team and that all information will remain confidential, and that no personal information will be used which may identify me or any other people in the final report or scientific publications.	
6. I understand that the echo results will be shared with my clinical care team including my GP where appropriate.	
7. I understand that data collected will be stored in secure University servers in line with current Data Protection regulations.	
8. I understand that data collected during the study may be looked at by individuals from regulatory authorities who have a duty to monitor the quality of the research, and I give permission for this.	
9. I agree to take part in this research study.	

Name of participant (capitals)	Date	Signature
_______________________	________	______________

Name of person taking consent (capitals)	Date	Signature
_______________________	________	______________
